# Antidromic Spike Propagation and Dissimilar Expression of P2X, 5-HT, and TRPV1 Channels in Peripheral vs. Central Sensory Axons in Meninges

**DOI:** 10.3389/fncel.2020.623134

**Published:** 2021-01-15

**Authors:** Oleg Gafurov, Kseniia Koroleva, Rashid Giniatullin

**Affiliations:** ^1^Department of Human and Animal Physiology, Institute of Fundamental Medicine and Biology, Kazan Federal University, Kazan, Russia; ^2^A.I. Virtanen Institute for Molecular Sciences, Faculty of Health Sciences, University of Eastern Finland, Kuopio, Finland

**Keywords:** migraine, meninges, trigeminal nerve, excitability, ATP, 5-HT, TRPV1 receptor

## Abstract

**Background:** The terminal branches of the trigeminal nerve in meninges are supposed to be the origin site of migraine pain. The main function of these peripheral sensory axons is the initiation and propagation of spikes in the orthodromic direction to the second order neurons in the brainstem. The stimulation of the trigeminal ganglion induces the release of the neuropeptide CGRP in meninges suggesting the antidromic propagation of excitation in these fibers. However, the direct evidence on antidromic spike traveling in meningeal afferents is missing.

**Methods:** By recording of spikes from peripheral or central parts of the trigeminal nerve in rat meninges, we explored their functional activity and tested the expression of ATP-, serotonin-, and capsaicin-gated receptors in the distal vs. proximal parts of these nerves.

**Results:** We show the significant antidromic propagation of spontaneous spikes in meningeal nerves which was, however, less intense than the orthodromic nociceptive traffic due to higher number of active fibers in the latter. Application of ATP, serotonin and capsaicin induced a high frequency nociceptive firing in peripheral processes while, in central parts, only ATP and capsaicin were effective. Disconnection of nerve from trigeminal ganglion dramatically reduced the tonic antidromic activity and attenuated the excitatory action of ATP.

**Conclusion:** Our data indicate the bidirectional nociceptive traffic and dissimilar expression of P2X, 5-HT and TRPV1 receptors in proximal vs. distal parts of meningeal afferents, which is important for understanding the peripheral mechanisms of migraine pain.

## Introduction

The typical pain signaling is based on the detection of the harmful stimuli by the peripheral nerve terminals of sensory neurons and *orthodromic* propagation of the nociceptive spike to the higher pain centers (Basbaum and Woolf, 1999; Millan, 1999; Julius and Basbaum, 2001). Much less is known about the opposite *antidromic* propagation of spikes from the spinal cord/brainstem to periphery. It was proposed that this phenomenon might take place in different body regions (Sorkin et al., 2018). However, in the trigeminal nociceptive system implicated in migraine pain, such antidromic (functionally opposite to orthodromic) propagation of spikes could play a very specific role important to specific mechanisms and clinical properties of this disorder. Thus, it has been proposed that the spikes generated at central branches of the trigeminal nerve or in the somas of the trigeminal neurons are propagated to the meninges (Geppetti et al., 2012). The role of these antidromic spikes propagated to the periphery, would be the control of the tone of local blood vessels, activation of mast cells and release from peripheral trigeminal nerve fibers of the neuropeptide CGRP, a main migraine mediator (Geppetti et al., 2012). These cellular and humoral factors are known as major contributors to activation and sensitization of the trigeminal nerve fibers in the meninges which are supposed to be the origin site of migraine pain (reviewed by Messlinger, 2009). Despite the multiple indirect effects described above the direct evidence of antidromic spike propagation by trigeminal nerve fibers in meninges is lacking.

There could be several trigger points for initiation of antidromic spikes: (1) other branches of the same axon (Sorkin et al., 2018); (2) central nerve terminals activated by GABA (Willis, 1999; Lin et al., 2007); (3) somas of the trigeminal neurons, expressing a wide range of receptors and cross-talking with satellite glial cells (SGCs) which control excitability of local neurons (Laursen et al., 2013; Omoto et al., 2015; Hanani and Spray, 2020; Messlinger et al., 2020).

Recently we showed that extracellular ATP, serotonin (5-HT), and capsaicin, operating via specific membrane receptors, are the powerful triggers of orthodromic spiking underlying meningeal trigeminal nociception (Zakharov et al., 2015; Yegutkin et al., 2016; Kilinc et al., 2017; Koroleva et al., 2019). However, it is unclear if these triggers can initiate the antidromic activity. In particular, it is unknown whether these axons express membrane receptors/pain transducers similar to those which initiate the orthodromic activity.

Therefore, in the current project, we recorded the electrical activity of the central part of the trigeminal nerve in the meninges and studied the expression of ATP, capsaicin, and 5-HT receptors in the most proximal parts of this nerve.

## Materials and Methods

### Animals

The experiments were performed with Wistar 4–6 weeks old male rats, which were used for the hemiskulls preparations. Rats obtained from the Animal Houses of the University of Eastern Finland or Kazan Federal University were kept in special cages in rooms with controlled temperature/humidity and 12-h light/dark cycle with food and water *ad libitum*. The experimental protocols were approved by the Local Ethics Committee of KFU (protocol No. 8 dated 05.05.2015) by the Committee for the Use of Animals of the University of Eastern Finland (license EKS-008-2019). All measures were taken to minimize the number of animals used in experiments.

### Meningeal Preparation and Solutions

The recording of orthodromic and antidromic spiking activity of meningeal nerves was performed in the isolated hemiskull preparation (Zakharov et al., 2015). In short, after cleaning the rat skull, it was divided into two hemiskulls. After recovery in artificial cerebrospinal fluid (aCSF), containing (in mM): 120 NaCl, 2.5 KCl, 2 CaCl_2_, 1 MgCl_2_, 11 glucose, 24 NaHPO_4_, 30 NaHCO_3_ with constant oxygenation of 95% O_2_/5% CO_2_, one of hemiskulls was placed in the recording chamber with continuous flow of aSCF. For activation of purinergic, serotonergic, and TRPV1 receptors, we diluted the agonists ATP, 5-HT, and capsaicin (Sigma–Aldrich, St. Louis, MO, USA) in aSCF. ATP and 5-HT were diluted in aCSF whereas capsaicin was diluted in DMSO (final concentration 0.1% which did not significantly change the spiking activity of the trigeminal nerve, *n* = 5, *p* > 0.05). These agonists were applied inside the hemiskull to meningeal areas around middle meningeal artery via gravity-driven perfusion system (speed 7 ml/min).

### Electrophysiological Recordings

**Figure 1:**
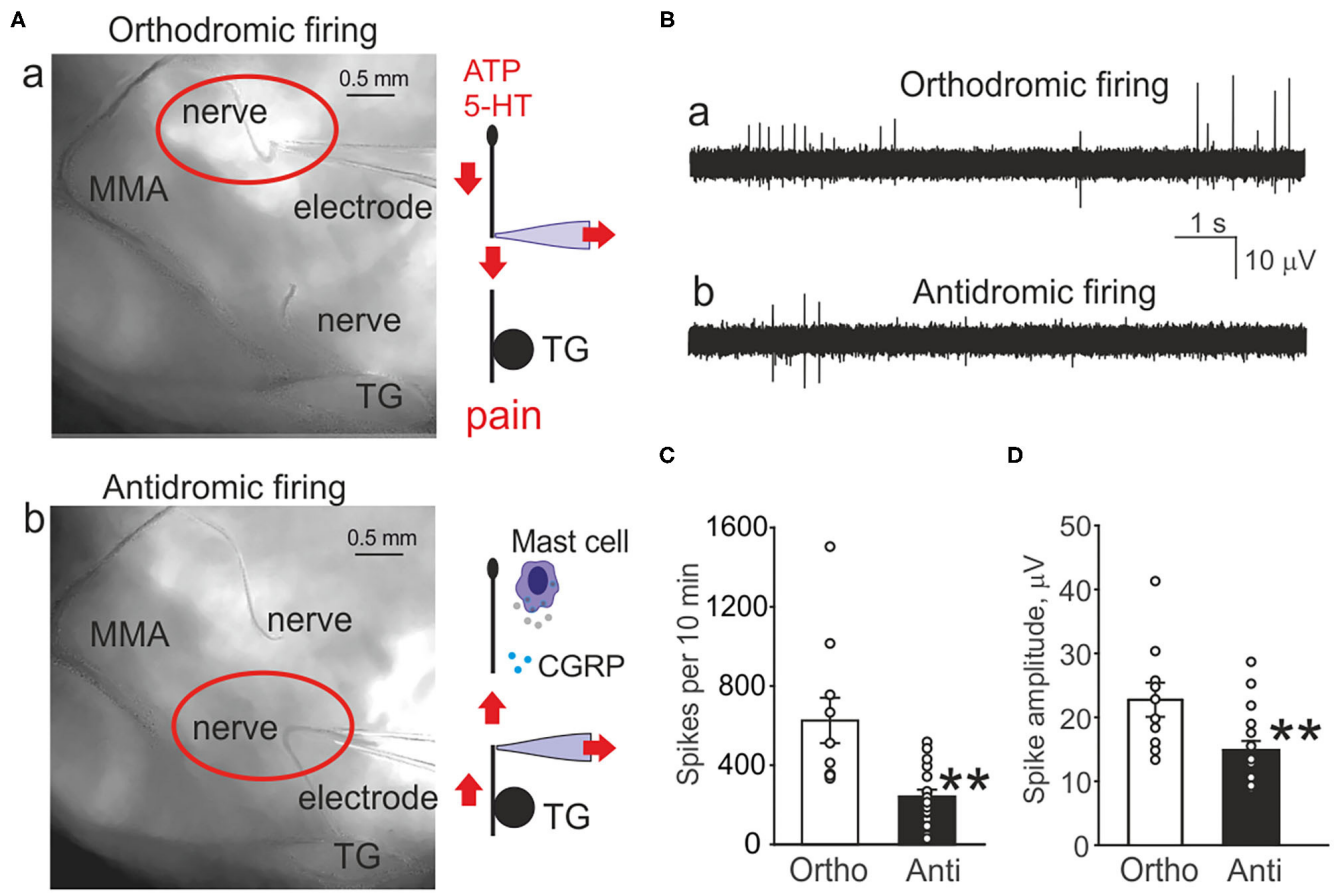
Orthodromic and antidromic propagation of action potentials in the trigeminal nerve. **(A)** Original images of the rat meninges with the middle meningeal artery (MMA), trigeminal ganglion (TG), and *N. spinosus* peripheral (a) or central parts (b) of the trigeminal nerve placed inside the recording electrode. Right, schematic presentation of the functional destination of the orthodromic and antidromic spike propagation. **(B)** Example traces of spontaneous action potentials propagated in the trigeminal nerve either in the orthodromic (a) and antidromic (b) directions. **(C)** Histograms showing comparison of the baseline frequency of orthodromic (Ortho, *n* = 10, white column) and antidromic action potentials (Anti, *n* = 17, black column) directions in the trigeminal nerve (**p < 0.01). **(D)** Histograms showing comparison of the baseline frequency of orthodromic (*n* = 10, white column) and antidromic action potentials (*n* = 17, black column) in the trigeminal nerve; mean ± SEM, **p < 0.01, Mann-Whitney test.

The orthodromic activity of the trigeminal nerve was recorded at stable room temperature from the peripheral nerve branch of *Nervus spinosus* (a branch of trigeminal nerve) after a cut of this nerve at the distance of ~1.5 mm from the trigeminal ganglion (Figure 1Aa red oval). The isolated end of the nerve branch was placed inside the glass microelectrode (for details, Zakharov et al., 2015). The antidromic activity was registered from the central part of this meningeal nerve in a separate hemiskull preparation (Figure 1Ab red oval). After successful suction of the nerve inside the electrode, the former completely plugged the tip of the latter prevented solution to get inside the electrode. In part of experiments, the proximal part of the nerve was disconnected from the trigeminal ganglion by cutting nerve at its entrance to ganglion. Electrical spikes were recorded with the DAM80 amplifier (World Precision Instruments, Sarasota, FL, USA), with gain 10,000 and bandpass 300–3,000 Hz and digitized with the NI PCI6221 board (National Instruments, Austin, TX, USA) using WinEDR v.3.2.7 software (Strathclyde University, UK).

### Cluster and Spectral Spike Analysis

For advanced spike analysis, we performed cluster and spectral analysis described by us earlier (Zakharov et al., 2015; Gafurov et al., 2017). At the preparatory stage, the experimental data were filtered using a Chebyshev filter type 2. To determine the threshold spikes searching, the baseline noise level was estimated for 12 s and the standard deviation (SD) was calculated. The spikes detection was made with a threshold of 4 SD of the baseline noise. Spike parameters were calculated using the MATLAB software package (MathWorks, Natick, MA, USA), such as: amplitude of the positive and negative phase, rise-time, decay time, spike areas and their total duration (Zakharov et al., 2015, 2016).

### Data Analysis and Statistics

Obtained data processing was performed using MATLAB and Origin Pro 2015 software (OriginLab, Northampton, MA, USA). The Student's *t*-test was used for paired samples and Mann–Whitney U-test for independent samples. Resulting data were presented as the mean ± standard error of the mean (m ± SEM). The difference was considered significant at *p* < 0.05.

## Results

### Comparison of Orthodromic vs. Antidromic Baseline Spiking Activity

Figure 1A shows the pictures of the rat hemiskull with the receptive field around middle meningeal artery and the branch of the trigeminal nerve (*N. spinosus*) innervating this area. The recording electrode contains either the peripheral part (Figure 1Aa red oval) or the central part of the nerve cut close to the trigeminal ganglion (TG) (Figure 1Ab red oval). The associated schematic presentation (Figure 1Aa, right) indicates the distinct functions of orthodromic firing (afferent nociceptive signaling) vs. antidromic activity (Figure 1Ab, right) (efferent control of CGRP release and mast cells activation). This scheme also shows the experimental approach for recordings from both sides of the cut nerve.

Figure 1B demonstrates the original traces of ortho- vs. anti-dromic baseline spiking activity with two modes of recording. Notice that the afferent orthodromic activity from the peripheral part (Figure 1Ba) was much higher than from the central part (Figure 1Bb) of the nerve.

In order to evaluate the spontaneous generation of spikes, we tested the baseline electrical activity recorded from the distal and proximal parts of the nerve (Figure 1C). Thus, the frequency of spikes recorded from the peripheral part of the trigeminal nerve was 626 ± 114 spikes per 10 min (*n* = 10, Figure 1C). Notably, spikes were recorded also from the central part of the trigeminal nerve. However, in contrast to peripheral orthodromic spiking, the firing rate of antidromic activity was significantly lower (242 ± 35 spikes per 10 min, *n* = 17, *p* = 0.001, Figure 1C). The mean amplitude of orthodromic spikes was 23 ± 2.7 μV (*n* = 10, Figure 1D) whereas, in antidromic activity, it was 15 ± 1.4 μV (*n* = 17, *p* = 0.008, Figure 1D).

In order to understand the origin of antidromic spikes, after obtaining the nociceptive firing in the hemiskull preparation, we disconnected the proximal part of the nerve from the trigeminal ganglion (Supplementary Figure 1, top scheme). This procedure first generated transient firing, which progressively declined by ~8 min to the stable very low level suggesting that ganglion contributed to the spontaneous nerve activity (Supplementary Figure 1A). Thus, to test the action of ATP on the disconnected nerve we first measured the basal activity in the period 8–10 min after cutting. The subsequent action of 100 μM ATP induced slight activation in this central part of the nerve, which, however, was not significant (*n* = 5, *p* = 0.09; Supplementary Figure 1B). Similarly, the subsequent action of 2 μM 5-HT was also non-significant (*n* = 5, *p* = 0.30; Supplementary Figure 1C). Notably, after ATP washout the basal activity was exceptionally low, just few spikes over 10 min period, giving *circa* 15-fold reduction in comparison with preparation with the preserved trigeminal ganglion. Application of 50 mM KCl at the end of this protocol produced a significant firing indicating the viability of the disconnected proximal nerve (Supplementary Figure 1A).

In summary, we obtained a direct evidence of the antidromic propagation of spikes, which frequency was several folds lower that electrical traffic from the periphery.

### Induction of Orthodromic vs. Antidromic Activity by ATP and 5-HT

**Figure 2:**
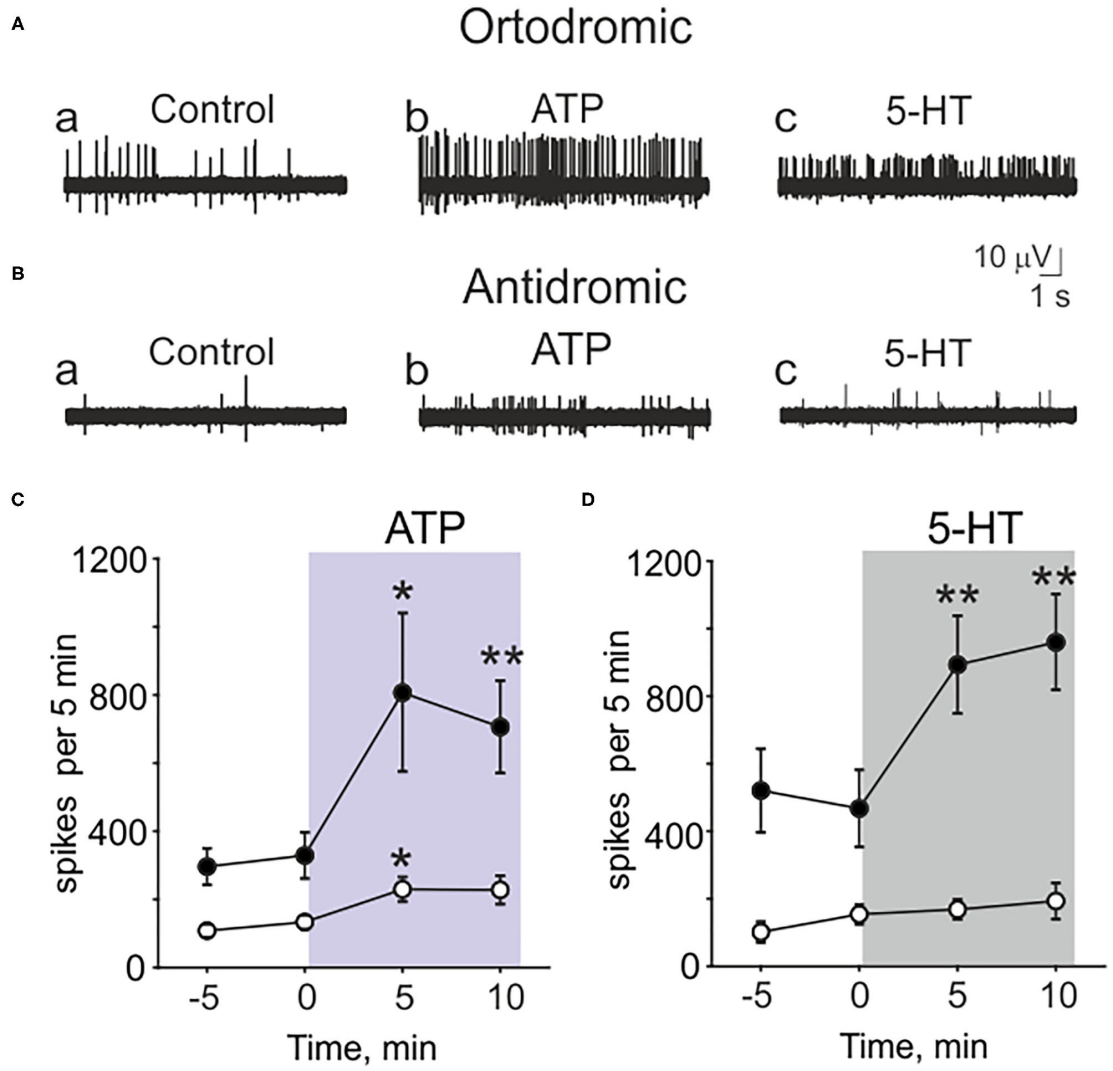
Testing the action of ATP and serotonin (5-HT) in peripheral (orthodromic) and central (antidromic) parts of trigeminal afferents. **(A)** Example traces of orthodromic action potentials in the trigeminal nerve in control (a), after application 100 μM ATP (b) and 2 μM 5-HT (c). **(B)** Example traces of antidromic action potentials in the trigeminal nerve in control (a), after application of 100 μM ATP (b) and 2 μM 5-HT (c). **(C)** The frequency of orthodromic (*n* = 10, black cycles) and antidromic (*n* = 17, white cycle) action potentials during application of 100 μM ATP. **(D)** The frequency of orthodromic (*n* = 10, black cycles) and antidromic (*n* = 10, white cycles) action potentials during application of 2 μM 5-HT; mean ± SEM, *p < 0.05, **p < 0.01, *t*-test.

Next, we compared the functional expression of pain transducers in central and peripheral parts of the trigeminal nerve in the meninges. To this end, we recorded the electrical activity either from the central or peripheral part of the trigeminal nerve after activation of ATP and 5-HT receptors. ATP (100 μM) was applied with high speed (7 ml/min) for 10 min and then washed out for 20 min. Next, 5-HT (2 μM) was applied for 20 min, followed by washout. At the end of some recordings, 1 μM capsaicin was applied (see below). Both ATP and 5-HT very efficiently increased the frequency of spikes recorded from the peripheral part of the trigeminal nerve. Thus, 100 μM ATP increased the spiking activity from 329 ± 68 to 807 ± 232 spikes per 5 min (*n* = 10, *p* = 0.029, Figures 2Aa,b,C black cycles). For the next 5 min, ATP changed activity to 706 ± 136 spikes (*n* = 10, *p* = 0.0009, Figure 2C black cycles). Similarly, 5-HT (2 μM) increased the neuronal activity from 468 ± 114 to 893 ± 145 spikes (*n* = 10, *p* = 0.00005, Figures 2Ac,D black cycles). For the next 5 min, 5-HT triggered 960 ± 141 spikes (*n* = 10, *p* = 0.0004, Figure 2D black cycles).

Recording from the central part of the trigeminal nerve, despite the low basal activity, ATP (100 μM) was still effective (Figures 2Ba,b,C white cycles). ATP increased the spiking activity in central part from 134 ± 21 to 230 ± 37 spikes per 5 min (*n* = 17, *p* = 0.027, Figures 2Ba,b,C white cycles). However, antidromic activity was apparently insensitive to application of 5-HT (Figures 2Bc,D white cycles). Thus, in control it was 154 ± 29 spikes per 5 min and after application of 2 μM 5-HT the frequency of spikes was almost the same (169 ± 30, *n* = 10, *p* = 0.686, Figures 2Bc,D white cycles).

These data indicated the different sensitivity to several agonists of main nociceptive receptors in peripheral vs. central parts of the trigeminal nerve.

### Cluster and Spectral Analysis of ATP and 5-HT Effects

**Figure 3:**
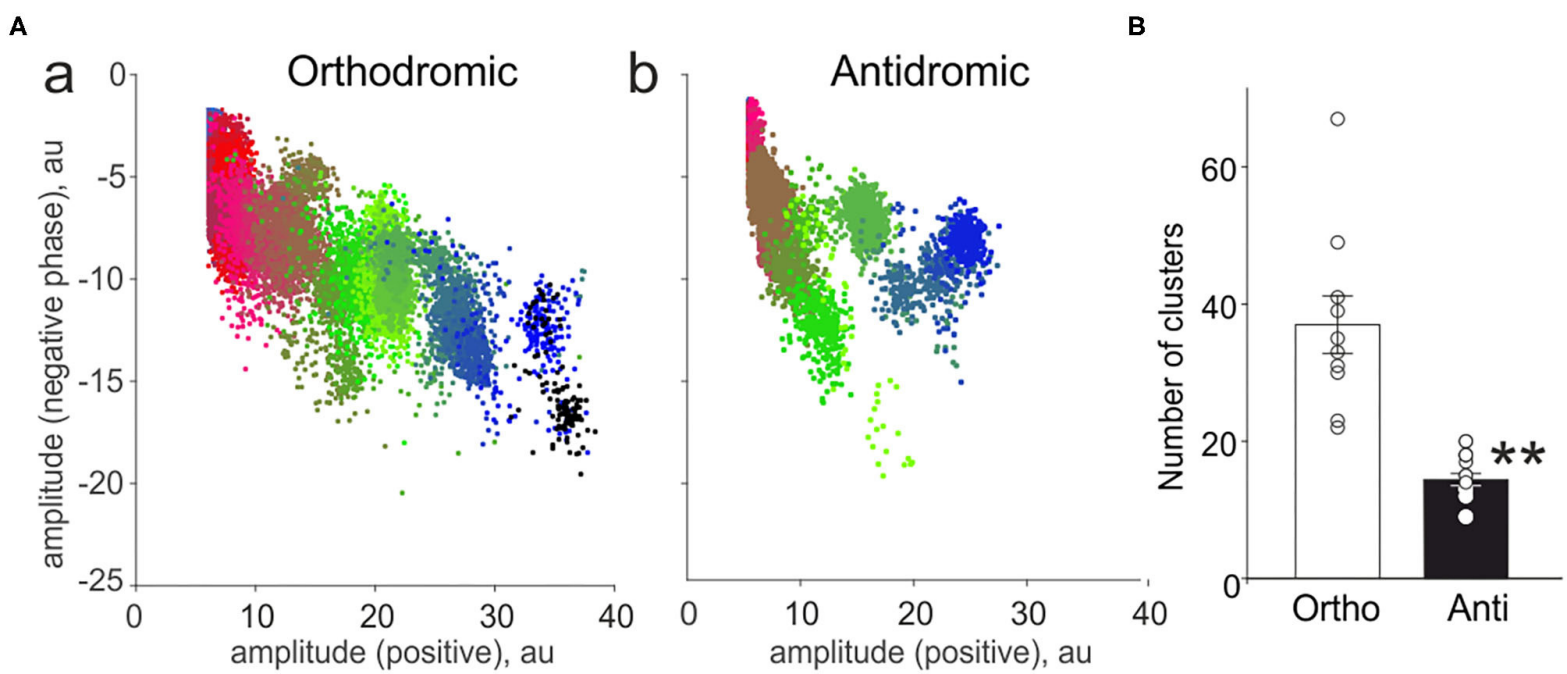
Cluster analysis of spiking activity in the trigeminal nerve in the orthodromic and antidromic directions. **(A)** The example distribution of clusters (plot of the amplitude of the negative spike phase vs. positive phase) in the orthodromic (a) and antidromic (b) firing. Notice a smaller number of clusters during antidromic firing. **(B)** Histograms showing comparison of the total number of clusters in the orthodromic (Ortho, *n* = 10, white column) firing and antidromic (Anti, *n* = 17, black column) firing; mean ± SEM, **p < 0.01, Mann-Whitney test.

In order to characterize the neurochemical profile of the nerve fibers constituting the peripheral and central part of the nerve, we applied the cluster analysis (Zakharov et al., 2015; Gafurov et al., 2017). In the peripheral part, we found 37 ± 4.2 functional clusters (*n* = 10, Figures 3Aa,B white column) representing activity of either single fibers, small groups of functionally similar fibers (Zakharov et al., 2015). In contrast, antidromic activity was presented only with 14 ± 0.9 clusters (*n* = 17, *p* < 0.0001, Figures 3Ab,B black column). These data indicated much higher number of active fibers contributing to orthodromic activity of the trigeminal innervation.

**Figure 4:**
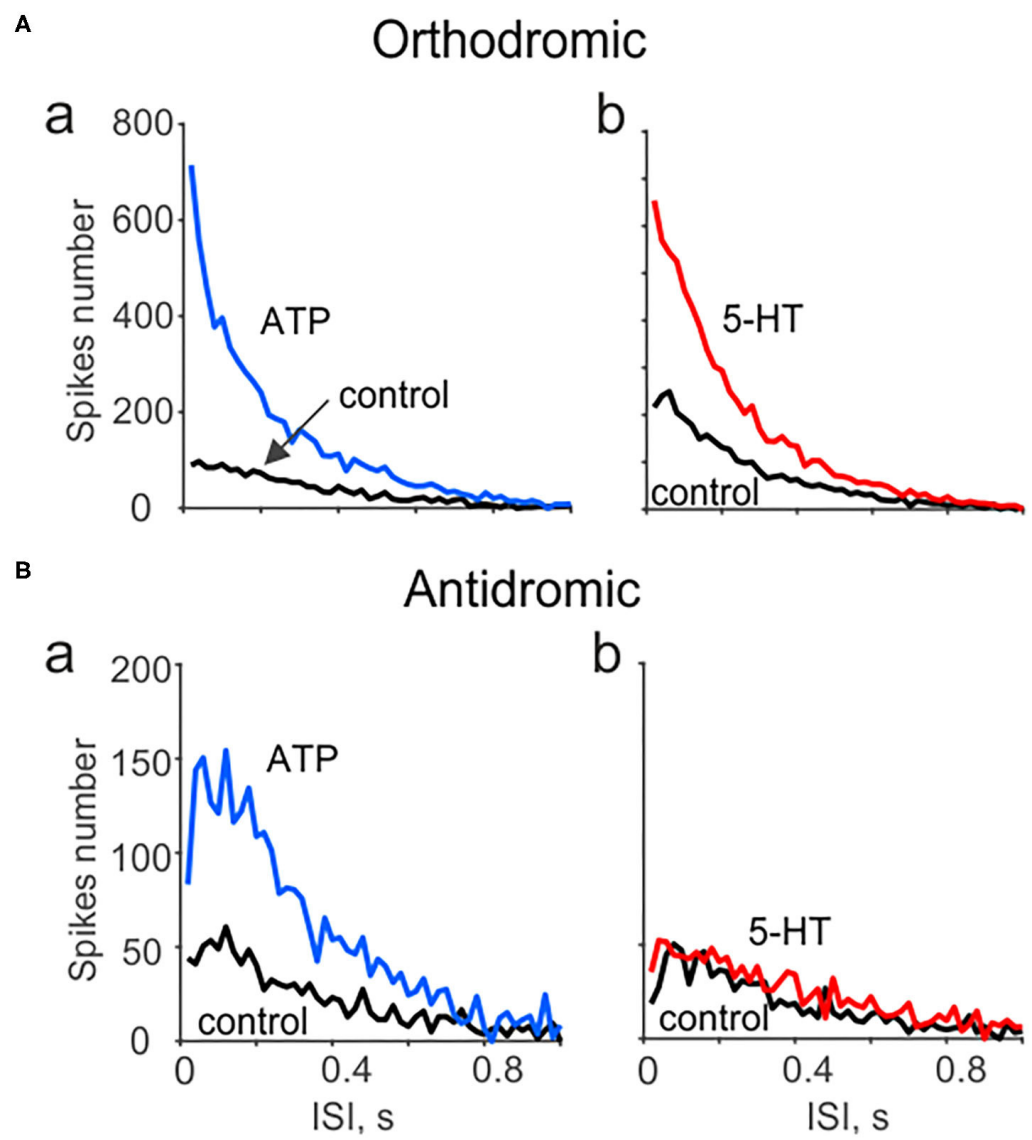
Spectral analysis of ATP and serotonin (5-HT) induced orthodromic and antidromic firing. **(A)** The distribution of interspike intervals (ISI) for orthodromic firing in control and after application of 100 μM ATP (a; *n* = 10) and 2 μM 5-HT (b; *n* = 10). **(B)** The distribution of interspike intervals (ISI) for antidromic activity in control and after 100 μM ATP [*n* = 17 (a) and for μM 5-HT (b, *n* = 10)]. Notice lack of changes in spectral distribution of antidromic spikes for 5-HT.

Moreover, we characterized the spectral properties of the neuronal activity in peripheral vs. central parts of the trigeminal nerve propagated in the opposite directions. Figure 4A shows that both ATP (a) and 5-HT (b) largely increased the nociceptive activity in the peripheral region, primarily in the range of very short interspike intervals (ISI). This suggests that these intense firing should efficiently amplify the transmission of arriving signals to the second order neurons, which relay the nociceptive traffic to the higher brains centers (Zakharov et al., 2015). Interestingly, an increase in nociceptive activity in the antidromic direction was observed only in response to ATP (although less efficiently than in peripheral part) but not to 5-HT (Figures 4Ba,b). This different coding of evoked activity fits well with the distinct functional destination of the orthodromic and antidromic firing (Figure 1).

### Stimulation of Orthodromic vs. Antidromic Activity by Activation of TRPV1 Receptors

As additional test for neurochemical profiling, we tested in both parts of this nerve the excitatory action of the specific agonist of TRPV1 receptors capsaicin. Previously we provided very detailed analysis of the potent excitatory effect of capsaicin on the peripheral part of meningeal afferents (Zakharov et al., 2015). Consistent with the previous data, 1 μM capsaicin increased the frequency of orthodromic spikes from 800 ± 173 to 2,677 ± 603 (*n* = 4; *p* = 0.033; Supplementary Figures 2Aa,C, black cycles). The main aim was, therefore, to test whether capsaicin can also activate antidromic spikes. Indeed, application of 1 μM capsaicin was highly effective in promoting spiking in the central part of the trigeminal nerve (increase from 234 ± 71 to 872 ± 121 spikes per 5 min (*n* = 10, *p* = 0.0009; Supplementary Figures 2Ab, C white cycles). Our experiments showed that capsaicin more significantly increased orthodromic than antidromic spiking activity (3,674 ± 778 vs. 1,252 ± 176 for 10 min, *p* = 0.024), respectively.

The data obtained indicated the expression of TRPV1 receptors not only in the nerve endings but also in the central part of the same nerves.

## Discussion

The main findings of this study are: (i) direct demonstration of the spontaneous antidromic activity of the trigeminal nerve in meninges essentially originating from trigeminal ganglion; (ii) spontaneous (tonic) and evoked activity of nerve is lower in central fibers compared to peripheral parts; (iii) presence of excitatory ATP and TRPV1 receptors in the proximal part of meningeal nerves; (iv) non-uniform distribution (proximal-distal gradient) of the serotonergic excitation in the trigeminal nerve, more prevalent at the periphery. Taken together, these data provide a novel description of the functionally distinct bidirectional nociceptive traffic in meningeal afferents implicated in generation of migraine pain.

The classical view implies that nociceptive signaling includes peripheral generation and orthodromic propagation of the spikes to the brain centers (Basbaum and Woolf, 1999; Millan, 1999; Julius and Basbaum, 2001; Gold and Gebhart, 2010; Giniatullin, 2020). The possibility of the functionally opposite, antidromic signal conduction in meningeal system was previously considered (Dimtriadou et al., 1991; Geppetti et al., 2012), but was based on indirect evidence. Thus, it was presumed that in the trigeminal nociceptive system, the antidromic spikes, generated at central terminals or in the somata of neurons, lead to the peripheral release of CGRP, dilation of meningeal vessels and degranulation of local mast cells (Geppetti et al., 2012). Notably, both CGRP and mast-cell derived pro-nociceptive molecules are contributing to activation of meningeal afferents and to the neurogenic inflammation, underlying the long-lasting pain in migraine (Theoharides et al., 2007; Levy, 2009; Olesen et al., 2009). Here, we show for the first time that there is a basal spike generation and their antidromic propagation, which, however, was less effective than the classical orthodromic nociceptive traffic from meninges toward the second order nociceptive neurons.

The most intriguing issues are: what is the natural trigger(s) for antidromic firing? Where are they produced? And are they amplified in migraine state? One potential triggering point for antidromic firing is the brainstem or spinal cord where the nociceptive primary afferent inputs are under the inhibitory GABAergic control. These sensory neurons contain an enhanced level of chloride ions leading to primary afferent depolarization (PAD) and dorsal root reflex (DRR) mediated by GABA (Vinay et al., 1999; Lidierth, 2006; Lin et al., 2007). Notably, PAD was so far only described for spinal and not meningeal afferents which, however, does not exclude the possibility of its generation in this part of the nociceptive system. It has been shown that the distribution of intracellular chloride (high in sensory neurons) enables the PAD to elicit spiking activity without compromising the inhibitory effect of GABA on primary afferents (Takkala et al., 2016). However, we used the hemiskull preparation, which contained trigeminal ganglia but was isolated from the brainstem/spinal cord. This makes it likely that spontaneous antidromic activity was mainly generated within the ganglion. Such conclusion is supported by our observation that the disconnection of the proximal part of the nerve from the trigeminal ganglion reduced spontaneous activity by almost 15-times. The trigeminal ganglion is a complex structure with multiple cell types, including sensory neurons surrounded by satellite glial cells (SGC), fibroblasts and immune cells such as macrophages (Franceschini et al., 2013). The chemical paracrine crosstalk between somas of the trigeminal neurons and SGCs could be a potential source of spikes propagated both in central and peripheral directions (Laursen et al., 2014; Omoto et al., 2015; Hanani and Spray, 2020; Messlinger et al., 2020).

The coding of neuronal signaling is known to be determined by the precise location of spike triggering points (Städele and Stein, 2016). Peripheral parts of primary afferents are equipped by a set of multiple membrane proteins/pain transducers, which are triggering the receptor potential leading to the spike generation (Giniatullin, 2020). Previous studies showed the leading role of TRPV1/TRPA1 receptors as important contributors to peripheral mechanisms of meningeal signaling (Messlinger, 2009). By direct recordings of meningeal spikes, we recently confirmed the widespread (up to 60%) expression of TRPV1 receptors in peripheral fibers of meningeal afferents (Zakharov et al., 2015), and proposed the role of TRPA1 (Shatillo et al., 2013), ATP (Yegutkin et al., 2016; Koroleva et al., 2019) and 5-HT receptors (Kilinc et al., 2017) as triggers of nociception at nerve terminals. These powerful algesic agents were used in this project to compare the presence of respective excitatory receptors in peripheral and central parts of the trigeminal fibers. Importantly, we found that ATP and capsaicin (but not 5-HT) were able to excite not only peripheral parts but also the central portion of the trigeminal nerve. Although we cannot exclude that these agents act at the somas of neurons relatively deeply buried within the ganglion, the axonal expression of P2X and TRPV1 receptors is consistent with several previous observations. Thus, the distribution of TRPV1 not only at fine peripheral terminals but also along unmyelinated axons is supported by findings that neuropeptide exocytosis was observed along the nerve segments (Bernardini et al., 2004). Moreover, it has been shown that capsaicin can depolarize isolated sciatic rat nerves (Hayes et al., 1984), dorsal roots (Ault and Evans, 1980) and when applied to axons, excite both Aδ and C-fibers (Such and Janc, 1986). Likewise, it has been shown that protons, known as TRPV1 agonists, induced CGRP release from the sections of nerve axons (Fischer et al., 2003). These data and our observations on the ability of ATP and capsaicin to generate antidromic spikes suggest the presence of respective receptors in the central part of the nerve. These spikes are expected to be propagated to periphery, irrespective of the natural direction in this nerve, thus contributing to evoked “antidromic activity.”

Discussing the origin of antidromic activity we should also consider other mechanisms. Thus, it could be that the evoked antidromic activity was generated by nerve fiber collaterals. However, such contribution is expected to be very low as the central part of the nerve in our study was very close to the ganglion whereas the axonal branching is most significantly presented at the periphery (Suleimanova et al., 2020).

In contrast to ATP and capsaicin, the action of 5-HT was not significant at the central part of the trigeminal nerve. This is consistent with observation that 5-HT strongly activates the peripheral nerve terminals in meninges *via cys*-loop 5-HT3 receptors, but this neurotransmitter exerts the inhibitory action on signal transmission from central nerve terminals to the second order spinal cord neurons (Kilinc et al., 2017). These data suggest a decreasing gradient of serotonergic excitatory effect from peripheral to the central part of the trigeminal afferents. Similar non-uniform spatial distribution within C-fiber axons has also been shown for the pro-nociceptive Nav1.8 calcium channels, which are more prominent in the most distal axons and peripheral nerve terminals (Klein et al., 2017).

The peripheral parts of the trigeminal neurons are functionally distinct in activity from the central terminals because they can form the neuro-immune synapse with mast cells, massively localized around MMA in the meninges (Levy et al., 2007; Kilinc et al., 2017; Giniatullin et al., 2019). These immune cells can release a plethora of pro-nociceptive transmitters including 5-HT (Kilinc et al., 2017), which can amplify the action of classical algogen ATP, acting *via* neuronal 5-HT3 receptors at nerve terminals (Koroleva et al., 2019; Suleimanova et al., 2020). In turn, release of ATP could be amplified by mechanical forces from pulsating meningeal vessels acting via neuronal or vascular Piezo channels (Cinar et al., 2015; Wang et al., 2016), further contributing to meningeal nociception (Mikhailov et al., 2019; Della Pietra et al., 2020; Suleimanova et al., 2020). The crosstalk between various pro-nociceptive factors in the receptive field of meninges around MMA can explain our current observation on why the peripheral parts of the trigeminal nerve are more effective nociceptive devices. Indeed, we found that the orthodromic spontaneous traffic is much more powerful than the antidromic basal activity.

In conclusion, we provide a direct evidence of the antidromic spike generation and propagation in meningeal afferents implicated in migraine pain. This antidromic electrical activity suggests a mechanistical explanation for the previously described phenomena in meninges proposed as key contributors of migraine mechanisms such as CGRP release, mast cells degranulation and local vascular effects. It would be interesting in future to explore antidromic activity in migraine models when this efferent spike flow most likely is elevated. One of potential mechanisms for such pain signal amplification could be the positive synergetic interaction between the orthro- and anti-dromic signaling. Thus, it has been shown that the antidromic stimulation increased the effectiveness of orthodromic activity via sensitization of C-fibers (Gong and Lin, 2019). Moreover, during inflammation, GABAergic PAD (proposed as generator of antidromic activity) is increased (Willis, 1999; Lin et al., 2007), leading to enhanced orthodromic spiking (Takkala et al., 2016). These non-canonical GABAergic excitatory mechanism along with increased intra-ganglion crosstalk between neurons and glia could result in migraine-related peripheral effects in the meninges such as enhanced CGRP release, vasodilation, mast cell degranulation, and associated local sterile inflammation (Messlinger, 2009; Della Pietra et al., 2020).

## Data Availability Statement

The raw data supporting the conclusions of this article will be made available by the authors, without undue reservation.

## Ethics Statement

The experimental protocols were approved by the Local Ethics Committee of KFU (protocol No. 8 dated 05.05.2015) by the Committee for the Use of Animals of the University of Eastern Finland (license EKS-008-2019).

## Author Contributions

OG contributed to data collection, analysis, and writing the manuscript. KK contributed to data collection, analysis, interpretation, and writing the manuscript. RG contributed to the study design and supervision, writing the manuscript, and the final editing. All authors approved the final version of the manuscript.

## Conflict of Interest

The authors declare that the research was conducted in the absence of any commercial or financial relationships that could be construed as a potential conflict of interest.
