# Supplementary material for: Antidromic Spike Propagation and Dissimilar Expression of P2X, 5-HT, and TRPV1 Channels in Peripheral vs. Central Sensory Axons in Meninges
Source: Front Cell Neurosci. 2021 Jan 15;14:623134. doi: 10.3389/fncel.2020.623134 (PMC7845021; doi:10.3389/fncel.2020.623134)
Supplement: Supplementary file 1 [file Data_Sheet_1.PDF]

Antidromic Spike Propagation and Dissimilar Expression of P2X, 5-HT and TRPV1 Channels in Peripheral versus Central Sensory Axons in Meninges

Gafurov O, Koroleva K, Giniatullin R

Supplementary Material

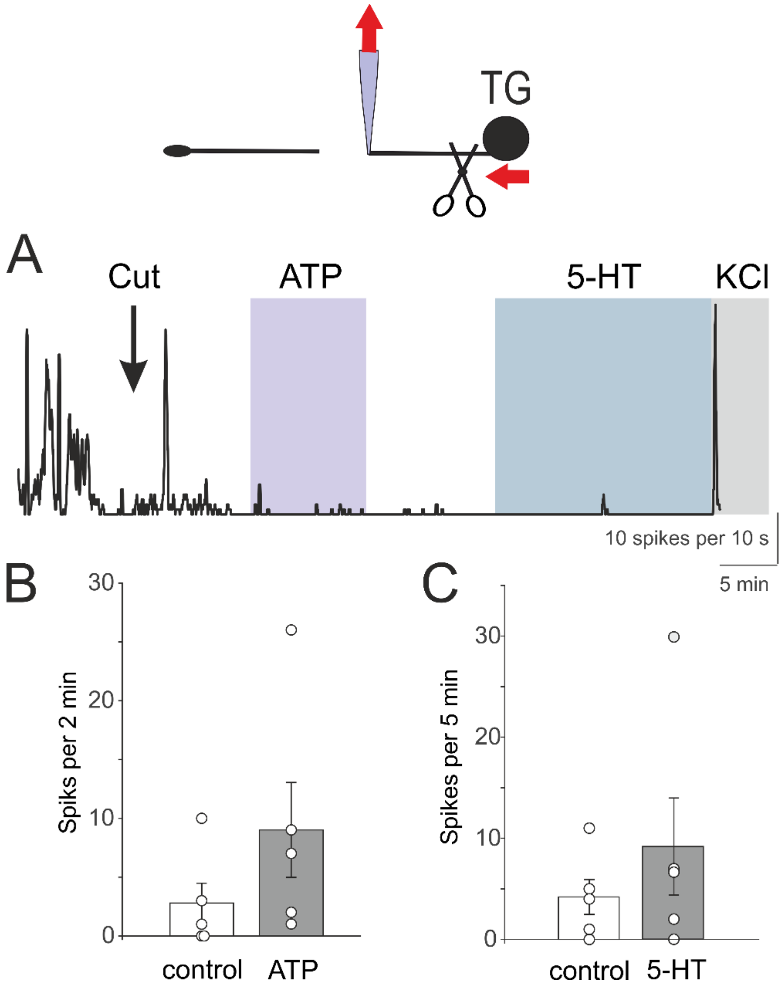

Figure S1. **The action of ATP and 5-HT on nociceptive firing in the proximal part of the trigeminal nerve after its disconnection with trigeminal ganglion (TG).** Top - scheme of experiment. (A) The time course of the changes in spiking activity in control, after nerve cut and after application of 100 µM ATP, 2 µM 5-HT and 50 mM KCl. Notice that KCl induced large firing indicating the viability of the nerve. (B) The histograms showing the number of spikes 2 min before and 2 min after application of 100 µM ATP (n=5; p=0.09). (C) The histograms showing the number of spikes 5 min before and 5 min after application of 2 µM 5-HT (n=5; p=0.3).

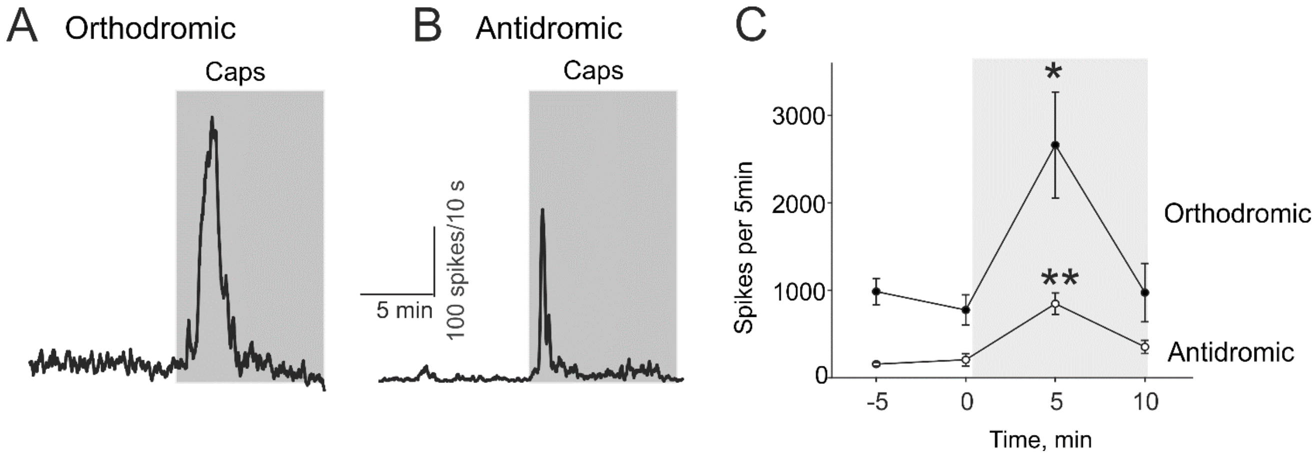

Figure S2. **The action of capsaicin in the peripheral (generating orthodromic firing) and central (antidromic firing) parts of trigeminal afferents.** (A) Example of orthodromic trigeminal nerve activity before and after application of 1 µM capsaicin. (B) Example of antidromic trigeminal nerve activity before and after application of 1 µM capsaicin. (C) The changes of frequency of orthodromic (n=4, black cycles) and antidromic (n=10, white cycles) action potentials during application of 1 µM capsaicin; *p <0.05; **p <0.01.
